# Profile of human anti-rabies care and post-exposure prophylaxis in the state of São Paulo

**DOI:** 10.1590/0037-8682-0473-2022

**Published:** 2023-03-27

**Authors:** Bruno Fonseca Martins da Costa Andrade, Luzia Helena Queiroz, Márcia Marinho

**Affiliations:** 1 Universidade Estadual Paulista “Júlio de Mesquita Filho”, Faculdade de Medicina Veterinária de Araçatuba, Programa de Pós-Graduação em Saúde Animal, Araçatuba, SP, Brasil.; 2 Universidade Estadual Paulista “Júlio de Mesquita Filho”, Faculdade de Medicina Veterinária de Araçatuba, Departamento de Produção e Saúde Animal, Araçatuba, SP, Brasil.

**Keywords:** Rabies, Disease notification, Post-exposure Prophylaxis, Bites

## Abstract

**Background:**

Rabies is an anthropozoonosis that greatly impacts public health and is transmitted by infected mammals. Aggression by animals is notifiable and may result in anti-rabies post-exposure prophylaxis (PEP). This study aimed to characterize anti-rabies PEP notifications in São Paulo state, Brazil.

**Methods:**

A descriptive study was conducted using data provided by the SINAN between 2013 and 2017.

**Results:**

A total of 572,889 aggressions were recorded during the study period, characterized mostly by dogs (83.5%), single wounds (56.9%), superficial wounds (58.6%), and hands/feet (34.6%).

**Conclusions:**

Animal observation was the most frequent recommendation, even in cases of attacks from non-domestic animals.

Rabies is an ancient anthropozoonosis with rapid and lethal progression. It is transmitted by contact with the neurotropic virus of the genus *Lyssavirus* of the *Rabdoviridae* family, which is disseminated through the saliva of the infected animal. Human infection occurs because of virus penetration in solutions of continuity in the skin or through the mucosa, where it reaches peripheral nerve endings, multiplies, and migrates to the central nervous system (CNS)^1^.

Human rabies can be prevented by prophylactic treatment, which is an important tool of the National Rabies Prophylaxis Program created in 1973 to guarantee disease control in Brazil. Rabies post-exposure prophylaxis (PEP) is based on the potential risk of infection by rabies virus, which is assessed by complete anamnesis using the Human Anti-Rabies Care Form of the Notifiable Diseases Information System (Sistema Nacional de Agravos de Notificação, SINAN)^1^. 

The correct characterization of aggression is necessary for proper adoption of rabies PEP recommendations following the guidelines of the Ministry of Health to prevent the victim from developing the disease, as well as to avoid the unnecessary application of either vaccine or rabies immunoglobulin when just observing the animal would be sufficient^2^ ^-^ ^5^.

Similar to other countries in America, Brazil has successfully controlled canine rabies and cases of human rabies transmitted by dogs and cats, thus reducing the risk of aggression in rabid dogs. Between 2010 and 2022 (August, 2), 45 cases of human rabies were registered in Brazil, and 76% of the cases were recorded in the North and Northeast regions. São Paulo is considered a geographical area with ​​controlled rabies, with no cases of human rabies transmitted by dogs recorded in this period^6^.

However, despite the success achieved both in Brazil and São Paulo, a high number of human anti-rabies treatments have resulted mainly from aggression by dogs^2^ ^,^ ^3^. To reduce unnecessary treatments, it is necessary to improve knowledge of the epidemiological profile of notified aggression cases. Studies involving aggression by dogs and cats have been conducted in the region of Araçatuba/SP to characterize the victim’s profile, type of injury, involved animals, and municipalities of notified occurrence^5^ ^,^ ^7^ ^,^ ^8^. This study aimed to describe the profile of human anti-rabies PEP after aggression by different animal species throughout the state of São Paulo between 2013 and 2017.

A descriptive study of the data reported in the compulsory notification form of human anti-rabies care, available on SINAN, was conducted for the established period (2013-2017) in all municipalities from the state of São Paulo. The incidence coefficient was calculated based on the human population estimate published by the Brazilian Institute of Geography and Statistics.

The information presented in the SINAN forms was made available by the Pasteur Institute, São Paulo, Brazil, after approval by the Research Ethics Committee of Faculty of Dentistry of the Universidade Estadual Paulista "Júlio de Mesquita Filho"/UNESP in Araçatuba, SP.

Statistical analysis of log-linear regression and Poisson distribution was performed to assess the annual percentage variation (APC) using the Joinpoint Regression Program (version 4.8.0.1, company, city, country), with a confidence interval (CI) of 95%. The spatial analysis was performed using ArcGIS 10.8 software (company, city, country).

During the study period, 572,889 anti-rabies post-exposure care were reported, with an annual average of 114,578 cases, 92.2% of which (528,208/572,889) occurred in urban areas. The search for anti-rabies PEP displayed an increasing trend that peaked in 2014, with an incidence of 2.62/1,000 inhabitants but was not statistically significant in the studied time interval (APC = 1.6%; 95% CI: -2.5 to 5.9; p = 0.307). During the period between 2014 and 2019, São Paulo had the highest number of human anti-rabies care notifications (708,307 [17.6%]), followed by Minas Gerais (438,500 [10.9%]), and Rio de Janeiro (RJ) (312,107 [7.7%])^2^. 

The incidence observed in our study was 2.52 cases per 1,000 inhabitants, a higher coefficient than the 1.22/1,000 inhabitants^9^ reported in Minas Gerais in 6 years and 0.85/1,000 inhabitants observed in Ceará in 9 years^4^. However, a study conducted with data from all over Brazil^3^ reported incidences similar to those found in São Paulo, with a coefficient of 2.57/1,000 inhabitants between 2008 and 2016, whereas Roraima had the highest coefficient (5.44/1,000 inhabitants) and Sergipe had the lowest (0.97/1,000 inhabitants).

São Paulo has the second highest number of municipalities in Brazil and is the most populous state in the country. Of the 645 municipalities, 17 had an incidence coefficient > 8.0 cases per 1,000 inhabitants. The incidence of notifications varied between the Regional Health Departments (DRS), but the difference was not statistically significant (APC = 2.0%; 95% CI: -1.3 to 5.4; p = 0.213). In municipalities of other states, the incidence coefficient also varied^10^ but no human anti-rabies care pattern was identified per region or population density, allowing us to conclude that aggression is a random accident arising from a reaction caused by the animal instinct^7^ ^,^ ^8^. 

The incidence of aggression was similar between the sexes, with a predominance in males; the highest frequency was in the age group between 20 and 39 years, and was more prevalent in white people (Table 1). The characteristics of the victims were similar to those reported in other studies^2^ ^,^ ^4^ ^,^ ^5^ ^,^ ^9^ ^,^ ^11^; however, a higher frequency of female victims^12^ was observed in RJ.

**TABLE 1:** Characterization of the anti-rabies post-exposure care (572,889) in the state of São Paulo between 2013 and 2017.

Characteristics	Occurrences	Percentage
Sex		
**Male**	298,435	52.09
Female	274,253	47.87
Unknown	201	0.04
**Age groups**		
0 to 12 years old	136,190	23.77
13 to 19 years old	57,449	10.03
20 to 39 years old	158,896	27.74
40 to 59 years old	135,844	23.71
> 60 years old	84,510	14.75
**Ethnicity/Color**		
White	346,829	60.54
Multiracial	101,076	17.64
Unknown	64,875	11.32
No information	28,876	5.04
Black	25,446	4.44
Asians	4,627	0.81
Indigenous	1,160	0.20
**Education**		
Illiterate	5,472	0.96
Elementary School	143,959	25.13
High School	107,177	18.71
University Education	47,678	8.32
Unknown	129,609	22.62
Not applicable	70,588	12.32
No information	68,406	11.94
Type of exposure*		
Bite	493,391	80.55
Scratching	76,030	12.41
Licking	15,570	2.54
Contact	8,819	1.44
Unknown	7,919	1.29
Other	7,649	1.25
No information	3,162	0.52
Injury site*		
Hands/feet	209,646	34.60
Lower limbs	199,310	32.90
Upper limbs	98,478	16.25
Head	51,532	8.51
Torso	22,576	3.73
Mucous	13,551	2.24
Unknown	10,763	1.78
**Wound**		
Single	325,699	56.85
Multiple	208,874	36.46
No wound	15,260	2.66
No information	12,798	2.23
Unknown	10,258	1.79
Type of wound*		
Deep	172,358	29.60
Superficial	341,305	58.61
No information	36,447	6.26
Tearing	28,465	4.89
Unknown	3,738	0.64

*Value above total notifications due to multiple injuries in a single victim. Source: Author, 2021.

When comparing sex and age groups, a predominance of notifications was observed among male children (27.8%; 83,107/298,435), while female victims between 20 and 39 years old were the most frequent (28.1%; 77,076/274.253). Occurrences predominantly in male children, female adults, and the elderly have been reported by other authors in different parts of Brazil^5^ ^,^ ^7^ ^,^ ^9^ ^,^ ^12^. 

Regarding education, the highest percentage of occurrences was observed in individuals with elementary school education at the time of aggression (Table 1), similar to data reported in Brazil (34.5%), where most of the occurrences were reported in individuals with complete elementary school education^2^. This predominance is possibly associated with the fact that lower-level education is more frequent among poorer individuals and, consequently, the dog is welcomed at home, not only as a pet but also to help protect the residence. The high percentage of forms with the information “unknown” in this field (22.6%) is also noteworthy. Similarly, in a study carried out in RJ, the education field had the highest rate of incompleteness^12^.

The canine species were predominant among aggressors, with 83.5% (478,082/572,889) of notified occurrences, followed by felines with 10.7% (61,164/572,889), whereas the other species together represented 1.5% (8,842/572,889) of notifications. The urban cycle (dogs and cats) represented 94.2% of aggressions, similar to those occurred in Brazil (96.7%) between 2014 and 2019^2^. The percentage of aggression by bats was only 0.8% (4,777/572,889), lower than the 1.4% value recorded in RJ^12^.

As for the type of exposure, there was a prevalence of biting, followed by scratching, totaling 92.9% of the reported aggressions. The injuries were predominantly single wounds and superficial, most frequently on the hands and feet, followed by the lower and upper limbs (Table 1). These results are similar to those previously reported in other studies conducted in municipalities in the region of Araçatuba^5^ ^,^ ^7^ ^,^ ^8^ and across the entire country^2^.

When correlating the aggression site and age, it was observed that injuries in children were more frequent in the lower limbs (25.5%; 38,025/149.310), head (23.4%; 34.871/149.310), and hands and feet (22, 2%; 33,221/149,310). In adulthood, aggression occurred more frequently on hands and feet (37.5%, 176,425/469,933), followed by lower limbs (34.3%, 161,285/469,933). The occurrence in children corroborates the fact that lesions tend to occur more frequently in anatomical regions of easy access (lower limbs and head), as the handling of animals results in attacks due to defense instincts. Lesions in adulthood, which occur predominantly on the hands and feet, probably occur during animal feeding when these areas are exposed and easily accessible^5^ ^,^ ^8^.

The characteristics of lesions caused by dog bites are similar to those reported in different studies in Brazil^2^ ^,^ ^4^ ^,^ ^5^ ^,^ ^7^, with a prevalence of single and superficial lesions originating from healthy animals, which differs from the notifications recorded in the rural region of Pernambuco, where the most frequent lesions were multiple and deep^10^, and in the city of RJ, where single and deep lesions^12^ were predominant. The characteristics of the identified lesions support the hypothesis that animal attacks are caused by a situation in which they feels threatened and acts on defense instinct^7^.

The most frequently adopted method of conduction was animal observation alone for 10 days (41.9%; 239,902/572,889), followed by prophylaxis with vaccine and animal observation (22.4%; 128.168/572,889). The highest prevalence of animal observation in the state of São Paulo differs from the prophylaxis adopted in the country as a whole between 2009 and 2013^13^, and animal observation and vaccination prophylaxis were the most frequent recommendation by other authors^11^ ^,^ ^12^. However, the animal observation recommendation requires a place in the city where the aggressor animal can stay for 10 days after the incident with the victim. The reduced frequency of animal observation recommendations reported in Brazil is identified as a deficiency of the surveillance system, as the animal is often characterized as "unobservable" due to the reluctance of professionals or the lack of health professionals to monitor the dogs^13^.

Among individuals attacked by species other than cats and dogs, conduct varied according to species (Figure 1). There was a 74.3%, 59.5% and 40.5% prevalence of serum + vaccination recommendation for bats, primates, and herbivores, respectively, whereas the vaccination indication was more frequent (56.9%) in individuals attacked by foxes. Furthermore, a study conducted in RS^8^ showed that in patients injured by herbivores, 81% were treated with serum and/or vaccine, which is much higher than the results observed in this study. In the group of individuals attacked by bats, primates, and foxes, treatment with serum + vaccine was indicated in 50% of cases^8^, lower than the percentage observed in our study. Results from Brazil in the period between 2014 and 2019 showed that serum + vaccination was indicated in 69% of the aggression cases by wild animals^2^.

**FIGURE 1:**
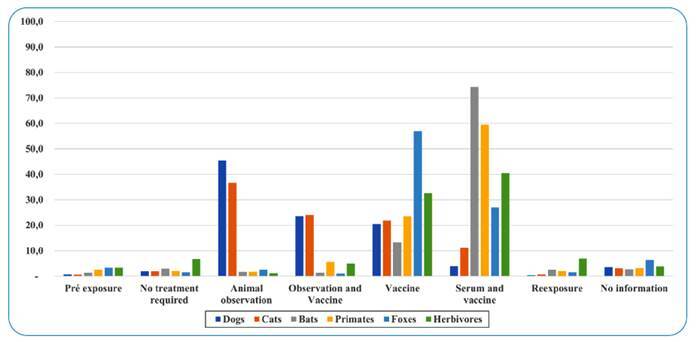
Percentage of treatments indicated as PEP against rabies in the state of São Paulo between 2013 and 2017.

Among the “no treatment required,” the highest percentage was for aggression by herbivores, which is justified because, often, the type of contact with these animals (consumption of raw milk), does not justify using either serum or vaccine. However, in our opinion, in aggression by bats, the protocol should be fully compliant, and the recommendation of both no-treatment and animal observation, as shown in Figure 1, is worrisome and demonstrates failure in the use of protocol, since in these cases the correct conduct is the use of serum and vaccine.

Most studies in Brazil regarding the indication of post-exposure anti-rabies treatments refer to aggression by dogs and cats, although none have reported on the frequencies of treatments indicated in the case of aggression by other species. In our study, when evaluating the distribution of individuals released from rabies prophylaxis due to attacks by species other than canines and felines, a higher prevalence was observed among the municipalities of DRS I (Greater São Paulo area), with more than 20% individuals who supposedly could be exposed to the risk of developing the disease (Figure 2).

**FIGURE 2:**
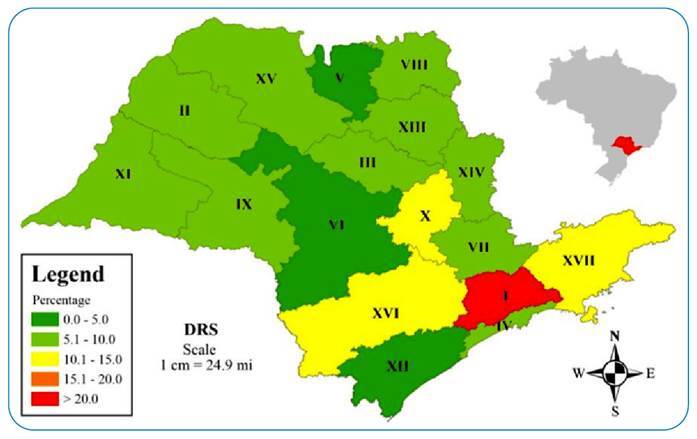
Distribution of “no treatment required” in individuals attacked by species other than canine and feline during PEP per DRS of São Paulo between 2013 and 2017.

In conclusion, the reported aggression rates in São Paulo were similar to the average rate observed in Brazil, and did not significantly differ between the studied Regional Health Departments. The most frequent aggression was from dogs, followed by cats and bats, and mainly affected male victims aged 20 to 39 years. Biting was the most frequent type of aggression, appearing as a single superficial lesion on the hands/feet, followed by the lower limbs. Animal observation was the most frequent recommendation followed by vaccine + observation. Among the accidents caused by other species, the highest percentage for using serum + vaccine was observed for bats, although in some municipalities, the frequency of no-treatment for individuals attacked by these species was higher than that observed in all the states.
